# Antimicrobial sensitivity of *Avibacterium paragallinarum* isolates from layers in the special region of Yogyakarta, Indonesia

**DOI:** 10.14202/vetworld.2021.1124-1127

**Published:** 2021-05-08

**Authors:** Ima Fauziah, Widya Asmara, Agnesia Endang Tri Hastuti Wahyuni

**Affiliations:** 1 Student of Doctoral Program of Veterinary Science, Faculty of Veterinary Medicine, Universitas Gadjah Mada, Yogyakarta, Indonesia; 2 Department of Microbiology, Faculty of Veterinary Medicine, Universitas Gadjah Mada, Yogyakarta, Indonesia

**Keywords:** antimicrobial susceptibility test, *Avibacterium paragallinarum*, infectious coryza

## Abstract

**Background and Aim:**

Infectious coryza (IC) is an upper respiratory disease of chicken caused by *Avibacterium paragallinarum*. Its clinical symptoms are swollen face and malodorous sinus exudate. This study was conducted to determine the antimicrobial sensitivity of *A. paragallinarum* isolates from layers in the Special Region of Yogyakarta, Indonesia.

**Materials and Methods:**

The samples used in this study were 30 layers that showed IC symptoms. The colony and cell morphology were observed with Gram staining; then, biochemical tests (catalase, oxidase, urease, indole, and motility tests, and carbohydrate fermentation tests using lactose, maltose, mannitol, and sorbitol) were performed to the suspected colony to identify *A. paragallinarum*. An antibiotic sensitivity test was performed using several antibiotic disks against *A. paragallinarum* isolates that were cultured on Mueller-Hinton Agar.

**Results:**

Out of 30 samples, 24 samples (80%) were found positive for *A. paragallinarum*. All isolates were sensitive to ampicillin (AMP) and amoxicillin (AML) (100%), and chloramphenicol (C) (91.6%). The antibiotics with intermediate sensitivity were enrofloxacin (79.2%), fosfomycin (75%), and ciprofloxacin (54.2%). The isolates were most resistant to erythromycin (100%), followed by tetracycline (87.5%), streptomycin (83.3%), doxycycline and kanamycin (70.8%), and trimethoprim (62.5%).

**Conclusion:**

Out of the total samples, 24 samples (80%) from layers with IC symptoms were identified biochemically as *A. paragallinarum*. It was sensitive to AMP, AML, and C.

## Introduction

*Avibacterium paragallinarum* is a Gram-negative bacterium that causes infectious coryza (IC), an upper respiratory tract disease in chickens [1,2]. The most common symptoms of IC are facial swelling, infraorbital edema, conjunctivitis, and malodorous nasal and ocular discharge [3]. IC occurs worldwide and causes great economic losses due to decreased egg production (up to 40%), increased culling rate of chickens, and increased medical costs [4]. *A. paragallinarum* can infect all types of chickens in multiage farms [5].

An appropriate selection and use of antibiotics for IC treatment must be considered because they can only reduce the clinical signs of IC but not cure the disease completely [6]. Various sulfonamides and antibiotics are useful in alleviating the severity and course of IC; however, no antibiotics have been found to be bactericidal. Erythromycin (E) and oxytetracycline are the two commonly used antibiotics [7]. *A. paragallinarum* has drug resistance, but there is no specific information about its resistance mechanisms [8].

Multidrug-resistant plasmid from *A. paragallinarum* isolates has been reported in Taiwan, where more than 75% of isolates were resistant to streptomycin (S), sulfonamides, kanamycin (K), and neomycin [9]. Relapse often occurs when the treatment is discontinued, and the carrier state is not eliminated [10]. Published data about the antimicrobial susceptibility of *A. paragallinarum* are limited. Therefore, antimicrobial susceptibility tests for *A. paragallinarum* are needed to determine the appropriate treatment.

This study aimed to determine the antimicrobial sensitivity patterns of *A. paragallinarum* isolates from layers in several farms in the Special Region of Yogyakarta, Indonesia.

## Materials and Methods

### Ethical approval

The samples were collected in accordance with standard collection procedure without hurting or necrotizing animals.

### Study period and location

The research was conducted from December 2018 to July 2019 in the Microbiology Department, Faculty of Veterinary Medicine, Universitas Gadjah Mada.

### Sample collection

The samples were collected from 30 layers with IC symptoms (malodorous nasal discharge and swollen face) and with no antibiotic treatment after infection. The layers were from several commercial layer farms in the Special Region of Yogyakarta and reared in a battery cage system. All samples were in the layer period (36-56 weeks) and vaccinated against IC.

### Isolation and identification of *A. paragallinarum*

The nasal exudate samples were cultured on a chocolate agar plate (Oxoid™, Basingstoke, UK), added with 5% sheep blood at 80°C, and incubated in an anaerobic jar for 24-48 h at 37°C. Identifications were made based on bacterial colony and cell morphology with Gram staining and biochemical tests (catalase test, oxidase test, urease test, motility test on semisolid media, indole test, and carbohydrate fermentation tests using lactose, maltose, mannitol, and sorbitol) [11-13].

### Antimicrobial susceptibility test

*A. paragallinarum* was cultured in brain heart infusion broth and incubated in an anaerobic jar at 37°C for 24-48 h. A bacterial suspension equivalent to 0.5 McFarland turbidity standard was made and spread onto Mueller-Hilton agar (MHA, Oxoid™). Antibiotic disks were placed on the agar surface. The medium was incubated in an anaerobic jar at 37°C for 24-48 h. Twelve antibiotic disks (Oxoid™) were tested: Amoxicillin (AML, 25 μg), ampicillin (AMP, 10 μg), chloramphenicol (C, 30 μg), trimethoprim (W, 5 μg), Erythromycin (E, 15 μg), tetracycline (TE, 30 μg), kanamycin (K, 30 μg), ciprofloxacin (CIP, 5 μg), streptomycin (S, 10 μg), fosfomycin (FOS, 50 μg), enrofloxacin (ENR, 5 μg), and doxycycline (DO, 30 μg). The antimicrobial sensitivity test was performed as recommended by the Clinical Laboratory Standards Institute [14], with some modifications by Chukiatsiri *et al*. [15]. The inhibition zone was observed, and the diameter was measured in millimeters. The susceptibility category (sensitive, intermediate, or resistant) was determined by comparing the zone of antibiotics with the zone diameter of *Escherichia coli* ATCC 25922 as the quality control strain [14]. For FOS, the inhibition zone category was based on the company that supplied the disk (Oxoid™).

## Results

In this study, 24 out of 30 samples (80%) were found positive for *A. paragallinarum*. The isolates showed tiny, circular, transparent, dewdrop-like Gram-negative coccobacilli colonies based on Gram staining. The isolates were non-motile, negative in catalase, oxidase, indole, and urease tests, and were able to ferment lactose, maltose, mannitol, and sorbitol based on biochemical tests (Table-1). The sensitivity test of *A. paragallinarum* to several antibiotics showed different results. All isolates were sensitive to AMP and AML (100%), and 22 isolates were sensitive to C (91.6%). The isolates were intermediately sensitive to ENR (79.2%), FOS (75%), and CIP (54.2%). The isolates were most resistant to E (100%), followed by TE (87.5%), S (83.3%), DO and K (70.8%), and W (62.5%). The sensitivity test results are shown in Table-2.

**Table-1:** The biochemical test results of the suspected isolates of *Avibacterium paragallinarum.*

Tests	Results	Positive isolates
Catalase	-	24
Oxidase	-	24
Urease	-	24
Indole	-	24
Motility	-	24
Lactose	+	24
Maltose	+	24
Mannitol	+	24
Sorbitol	+	24

**Table-2:** The results of sensitivity test of *Avibacterium paragallinarum* isolates from layers against 12 antibiotics.

Antibiotics (µg)	Zone diameter (mm)		R		I		S	
			
	S	R	n	%	n	%	n	%
A*moxicillin* (25)	≥18	≤13	0	0	0	0	24	100
Ampicillin (10)	≥17	≤13	0	0	0	0	24	100
Chloramphenicol (30)	≥18	≤12	2	8.3	13	54.2	9	91.6
Ciprofloxacin (5)	≥21	≤15	1	4.2	1	4.2	22	37.5
D*oxycycline* (30)	≥19	≤14	17	70.8	2	8.3	5	20.8
Enrofloxacin (5)	≥23	≤16	0	0	19	79.2	5	20.8
Erythromycin (15)	≥16	≤10	24	100	0	0	0	0
Fosfomycin (50)	≥18	≤11	1	4.2	18	75	5	20.8
Kanamycin (30)	≥18	≤13	17	70.8	7	29.2	0	0
Streptomycin (10)	≥21	≤14	20	83.3	3	12.5	1	4.2
Tetracycline (30)	≥23	≤13	21	87.5	3	12.5	0	0
Trimethoprim (5)	≥16	≤10	15	62.5	0	0	9	37.5

## Discussion

The treatment for IC has not been widely studied in Indonesia. There are few reports available about the susceptibility of *A. paragallinarum* isolates to antibiotics in Indonesia [13,16,17]. A total of 30 layers from several farms in the Special Region of Yogyakarta, Indonesia, were used as samples. They showed clinical symptoms, such as infraorbital sinus swelling, malodorous nasal exudate, and decreased egg production, as reported in the previous studies [18-20]. The nasal exudates were cultured on a chocolate agar plate. The colony morphology of 24 isolates showed round, small, transparent, and dewdrop-like colonies, which is similar to the previous studies [13,19,21]. Gram staining was done on 24 isolates and showed Gram-negative coccobacilli. The results are similar to the findings of Patil *et al*. [22] and Deshmukh *et al*. [23]. Then, the isolates underwent biochemical tests, such as catalase, oxidase, urease, indole, motility, and carbohydrate fermentation tests.

A total of 24 isolates identified as *A. paragallinarum* showed negative results in catalase, oxidase, indole, motility, and urease tests, which were also reported in the previous studies [11,15,16,20]. All isolates were able to ferment lactose, maltose, mannitol, and sorbitol, and similar results were reported in other studies [19,21,22]. Antimicrobial sensitivity test was done on isolates identified as *A. paragallinarum*. Antimicrobial sensitivity was determined by disk diffusion method.

The sensitivity test results of *A. paragallinarum* showed that the isolates were susceptible to several antibiotics at various levels. All isolates were sensitive to AMP, AML, and C. According to Akter *et al*. [5], Wahyuni *et al*. [13], and Han *et al*. [24], *A. paragallinarum* had the highest sensitivity to AMP and AML. *A. paragallinarum* isolates were found to be sensitive to penicillin and few penicillin groups of compounds, such as AMP and AML [23]. Luna-Galaz *et al*. [25] also reported that all reference isolates used in the study were sensitive to AMP and AML. The sensitivity to C was consistent with the previous studies [26,27].

The isolates had intermediate sensitivity to ENR, FOS, and CIP. Mohammad *et al*. [27] also reported that *A. paragallinarum* had intermediate sensitivity to CIP. Other studies reported that *A. paragallinarum* was sensitive to CIP and ENR [28]. Sensitivity toward FOS has not been described in any studies.

*A. paragallinarum* had the highest resistance level against E, followed by TE, S, DO, K, and W. Most of *A. paragallinarum* isolates have shown resistance primarily to sulfonamides and partly to aminoglycosides (especially S) and TE [23]. The resistance level against E was similar to other studies [9,15], whereas the high level of resistance against TE was similar to the study reported by Thenmozi and Malmarugan [29], and Heuvelink *et al*. [30]. Luna-Galaz *et al*. [25] reported that the Ecuadorian isolates were significantly more resistant to K and TE than the Mexican isolates. Resistance to TE and S was similar to other studies [8,31]. Poernomo *et al*. [16] reported that *A. paragallinarum* isolates were resistant to E, neomycin, S, and DO. The resistance to K and W was also reported by Wahyuni *et al*. [13]. Anjaneya *et al*. [32] reported that *A. paragallinarum* isolates were resistant to TE and DO, with no zone of inhibition.

## Conclusion

Out of the total samples, 24 isolates (80%) were identified as *A. paragallinarum*. The sensitivity test results show that *A. paragallinarum* isolates from layers were sensitive to AMP and AML (100%), and C (91.6%).

## Authors’ Contributions

AETHW: Planned and designed the study. WA: Contributed to the design of research and sampling. IF: Conducted the research and analyzed the results. IF: Prepared the manuscript under the guidance of AETHW and WA. All authors have read and approved the final manuscript.
